# Bouveret Syndrome: A Rare and Often Fatal Form of Gallstone Ileus

**DOI:** 10.7759/cureus.40005

**Published:** 2023-06-05

**Authors:** Mohamed Ibrahim, Ali El-Husari, Hozaifa Tabbaa, Michael Herman

**Affiliations:** 1 College of Osteopathic Medicine, Lake Erie College of Osteopathic Medicine, Bradenton, USA; 2 Gastroenterology, Borland Groover, Jacksonville, USA

**Keywords:** esophagogastroduodenoscopy (egd), bouveret's syndrome, exploratory laparoscopy, biliary tract fistula, gallstone ileus

## Abstract

Bouveret syndrome (BS) is a rare but serious complication of gallstone ileus that can cause gastric outlet obstruction secondary to a gallstone impacted in the pylorus or proximal duodenum. Gallstones pass from the gallbladder to the GI tract via a cholecystoenteric fistula that forms as a result of chronic inflammation and adhesions between the biliary system and GI tract. Although the case we are presenting is of a 53-year-old Hispanic male, females and the elderly are particularly at an increased risk of this condition. BS can present as typical mechanical obstruction symptoms that include nausea, vomiting, and diffuse abdominal pain. The vague symptoms patients present with makes the diagnosis difficult and often delayed, which can be fatal. In our case, the diagnosis of BS was supported by a CT with contrast, MRI, and an esophagogastroduodenoscopy (EGD) study. Our patient underwent an exploratory laparotomy after the diagnosis was made, and the stone was removed. Here, we aim to raise awareness of the importance of early recognition, and immediate action in establishing an early diagnosis of BS in patients presenting with nonspecific abdominal symptoms, which can prevent mortalities.

## Introduction

Bouveret syndrome (BS) is a very rare type of gallstone disease, making up only 2-3% of all gallstone-related intestinal obstructions, which by itself accounts for only 1-4% of all small bowel blockages [1]. It typically arises from a spontaneous cholecystoenteric fistula, connecting a diseased gallbladder to the distal stomach or proximal duodenum and ultimately causing a severe gastric outlet obstruction [2]. With only a reported 315 cases in the 50 years between 1967 and 2016, the management and treatment of this complication remain unstandardized [3]. Despite the rarity, mortality rates are surprisingly high at 12-30%, and it is heavily due to the late age of onset and presentation [2]. The prevalence of this disease is greatest among elderly women, with a median age of 74 years old and a female-to-male ratio of 1.9 [1]. This prejudice toward women is due to the commonly believed theory that sex hormones and ovarian stimulation during pregnancies cause an increased release of estrogen, which is known to increase biliary secretion of cholesterol causing saturation of the bile and gallstones [4]. This increased risk is not seen in males due to the lack of estrogen. Patients commonly present with intermittent episodes of nausea and vomiting, abdominal distention, and diffuse abdominal pain, while other more chronic patients may even experience dehydration and weight loss [2]. While there is no acceptable gold standard to manage these obstructions, various methods include more aggressive measures like laparoscopy or laparotomy or minimally invasive measures like endoscopy as well as laser, mechanical, electrohydraulic, or extracorporeal shock wave lithotripsy [3]. In this case report, we present a relatively young male who presented to the emergency room with complaints of abdominal pain, nausea, and vomiting for two days. After a complete workup, he was found to have a 6 cm by 3 cm gallstone in the distal body of the stomach that was obstructing his gastric outlet. He underwent an exploratory laparotomy, and the stone was successfully retrieved. The uniqueness of this case comes from the fact that it was a comparatively young male with no significant past medical history or risk factors.

## Case presentation

A 53-year-old Hispanic gentleman presented to the emergency room with complaints of abdominal pain, nausea, and vomiting lasting two days. After dinner, two nights before coming to the emergency room, he started noticing upper abdominal discomfort and heartburn sensations. Throughout the night, he felt extremely nauseous. He continued to notice the discomfort of the burning sensation up into the mid-central portion of the chest, which would radiate into the epigastric region upon burping. After he continued to have the symptoms, he could not sleep well. He did force himself to vomit, which made him feel much better. He had a good night's sleep and went to work the next day with no issues. Later in the evening, he started having the same sensation again. His upper abdominal pain was starting to get worse. He started vomiting again. He could not get comfortable throughout the night and into the early morning until he finally came to the emergency room around 3 AM. He denied having any fever, chills, dark stools, bloody stools, diarrhea, problems voiding urine, cough, chest pain, shortness of breath, burning or pain with urination, new onset of skin rashes, headaches, blurred vision, or muscle weakness. There were no sick contacts at home and no recent trauma to the abdomen. He was later consulted by gastroenterology after the emergency room stabilized him and ruled out any acute conditions.

Upon consultation with a gastroenterologist, the patient claimed he had been taking an oral supplement of iron for the past few months due to a previous history of chronic anemia. He said that he ate a quesadilla and salsa a few days prior and later had heartburn with reflux symptoms which were worse with laying down. He denied similar symptoms before, and the symptoms occurred multiple times over the next couple of days. He eventually vomited and noted phlegm and a dark brown liquid output. He denied bloody vomitus or black vomitus. He had been vomiting for about 20 minutes after eating anything, which got progressively worse. The patient stated he began to feel lightheaded and presented to the hospital for further evaluation. He denied any changes in bowel habits and continued to have a bowel movement daily, which was dark brown in color since starting his oral iron. He had a prior history of appendectomy but denied prior cholecystectomy or other abdominal surgery. He denied a family history of stomach cancer but said that his father had stomach ulcers and that his mother and two sisters had gastroesophageal reflux. He never had an upper endoscopy or colonoscopy before.

**Figure 1:**
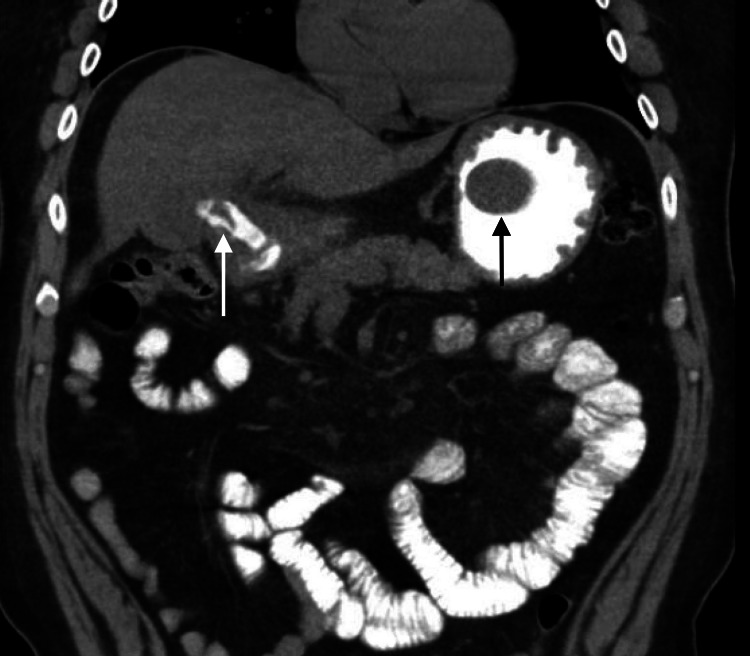
Coronal CT of the abdomen showing contrast crossing a cholecystoduodenal fistula (white arrow) and a 6 x 3-cm gallstone in the lumen of the stomach (black arrow)

**Figure 2:**
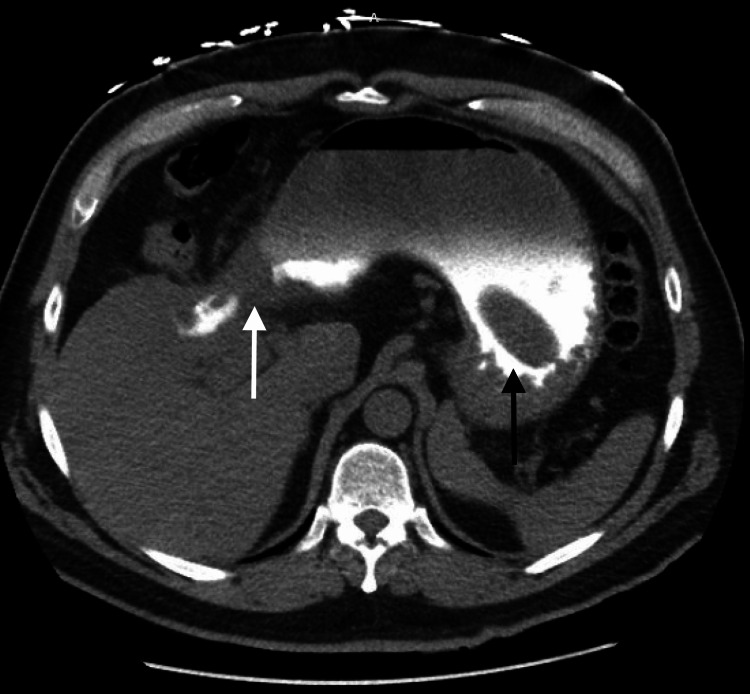
Axial CT of the abdomen showing contrast crossing a cholecystoduodenal fistula (white arrow) and a 6 x 3-cm gallstone in the lumen of the stomach (black arrow)

His physical exam and laboratory values were unremarkable except for some mild tenderness in the epigastrium with no guarding or rebound upon palpation. A CT with intravenous contrast of the abdomen and pelvis was performed in order to visualize the vasculature and soft organs. There were no acute abnormalities in the vasculature, but there was an abnormality involving the duodenum and what appeared to be the gallbladder. The appearance was unusual and suggested a possible fistula between the gallbladder and duodenum, which could have been secondary to a contained duodenal perforation and/or acute cholecystitis as seen in Figure 1 and Figure 2 below. The contrast also highlighted a 6 x 3 x 3 cm circumscribed oval structure in the lumen of the stomach which can also be seen in Figure 1 and Figure 2 below.

**Figure 3:**
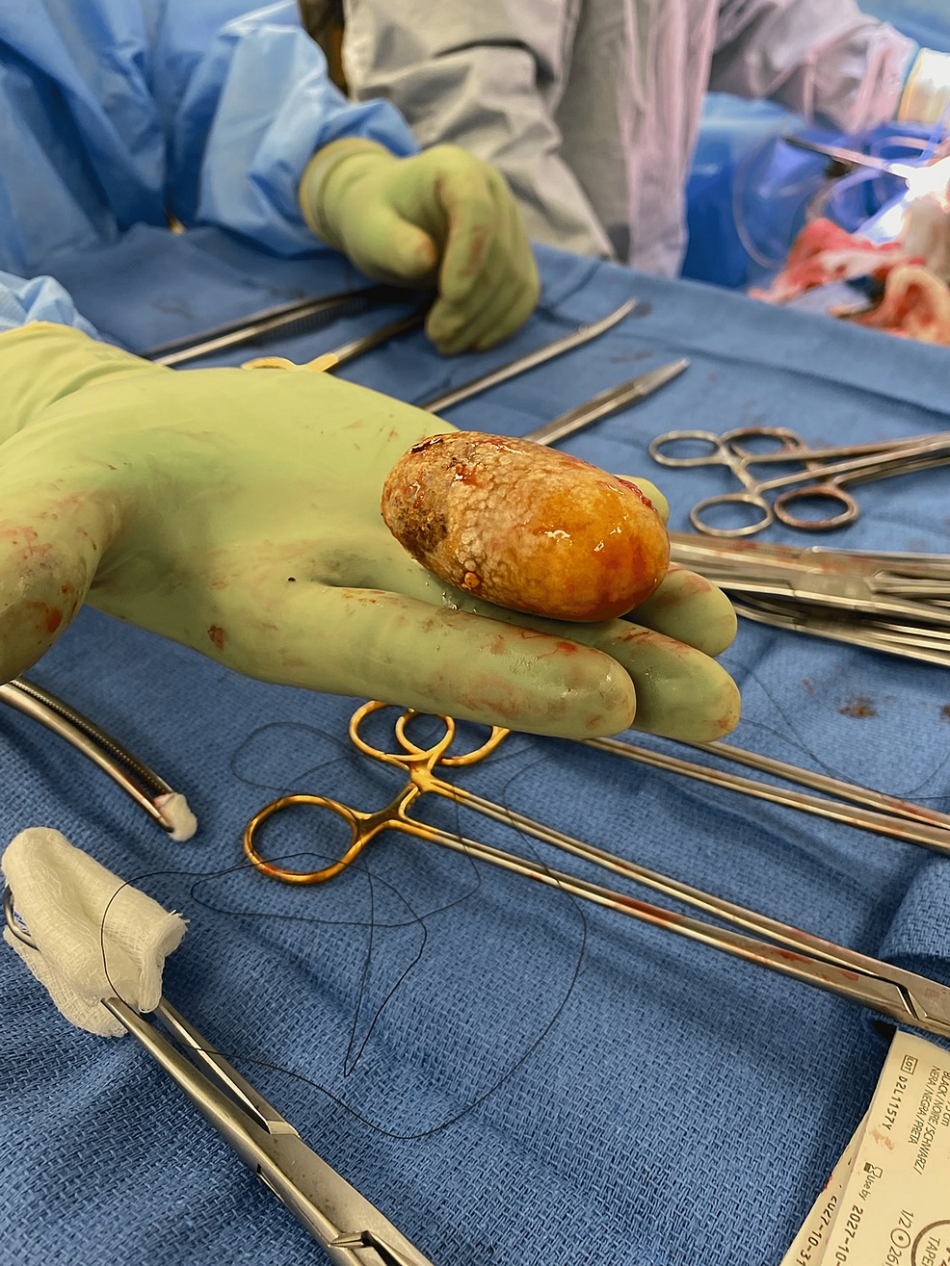
Postoperative retrieval of 6 x 3 cm gallstone from the distal body of the stomach

A surgical consultation was advised and an MRI of the abdomen without intravenous contrast was ordered to further evaluate the abnormal CT findings. The MRI confirmed the suspected irregularity and showed what appeared to be a fistulization from the gallbladder to the first portion of the pyloric/duodenal region with potential stones. This connection appeared to be associated with a contained duodenal perforation that measured 3.8 x 1.8 cm. An ovoid structure was also noted within the gastric lumen that may have represented a foreign body or bezoar. An esophagogastroduodenoscopy (EGD) study was performed to really evaluate and visualize if there was a stone and fistula. Upon completion of the EGD, a cholecystoduodenal fistula and a retained gallstone within the stomach were confirmed. Given the clinical presentation, CT findings, MRI findings, and EGD, a diagnosis of BS was established. An exploratory laparotomy surgery with cholecystectomy, gastrotomy, extraction of gallstone, pyloric exclusion, gastrojejunostomy, and repair of duodenotomy was planned and performed to fix the malformation. It was recommended that the large gallstone within the stomach be removed as this was the cause of gastric outlet obstruction. During the surgery, a gallstone that measured 6 cm in length and 3 cm in diameter was retrieved from the lumen of the distal stomach, and the cholecystoduodenal fistula was repaired through a gastrojejunostomy without any complications. An image of the gallstone postoperatively is shown below in Figure 3. The patient did well postoperatively and had no complaints from the operation.

## Discussion

BS usually presents with non-specific symptoms and relies on a combination of clinical suspicion and imaging for diagnosis [1]. The symptoms most commonly associated with BS are a triad of epigastric pain, nausea, and vomiting [1]. These symptoms can be accompanied by abdominal pain, distention, upper gastrointestinal bleeding, fever, weight loss, and anorexia [1]. CT is the preferred diagnostic imaging, while the most common imaging technique, X-ray, is only diagnostic in 21% of cases [1]. According to one report by Capell et al. [5] in 2006, CT is only diagnostic in 60% of cases. Though our patient fits these “typical” criteria, the patient himself was not the classic patient.

BS is most commonly found in the elderly female population [1,3,5]. According to Capell et al., the average age upon studying more than 100 cases was 74.1 +/- 11.1 (SD) years old with a female-to-male sex ratio of 1.86 [5]. Another report by Jin and Naidu also identified common risk factors as shown in Table 1 [3]. Our patient is unique in the fact that he did not fit any of the usual criteria for BS. Upon further literature search, it was seen that Hispanic, specifically Hispanic Americans, are at higher risk of gallstone formation than other races in the United States [6-9]. Certain genetic factors could explain the formation of gallstones in a Hispanic individual with no apparent risk factors [8]. It is also possible that some factors such as obesity, quick weight loss, medication use, or diet were not taken into account when assessing this patient's risk [9].

**Table 1:** Risk factors of BS Adapted from Jin and Naidu [3]

Risk factors for BS
Old age (>60 years old)
Female gender
Gallstones larger than 2 cm
Recurrent biliary colic and chronic cholecystitis
Post-surgical modified gastrointestinal anatomy

Treatment options for BS can be generalized into surgical intervention and minimally invasive procedures. Surgical techniques are often more favored due to their higher success rates and include gastric or enteral lithotomy through laparoscopy or laparotomy as well as repair of the fistula between the gallbladder and duodenum or the common bile duct and duodenum [3]. A cholecystectomy is also often performed to decrease the risk and source of complications in the future. The surgical options can be divided into two separate procedures, which is to retrieve the gallstone and repair the faulty connection while the other is to completely remove the gallbladder [3]. While this choice has fewer post-operative complications, the singular surgery is preferred due to lower rates of recurrent retrograde biliary sepsis and gallstone ileus [3]. Minimally invasive alternatives like endoscopic removal and shock-wave lithotripsy provide low-risk possibilities for those who cannot undergo the rigors of open surgery, but their success rates are only 10-25%, making surgery the optimal choice for complete resolution [3].

This paper was limited by the emergent presentation of the patient. The patient had not been going to a medical professional which limited information on the patient's health baseline. It would have also been useful to be able to perform some genetic tests to rule in or out factors that may have predisposed the patient to the formation of gallstone, such as being Native American or having the adenosine triphosphate binding cassette subfamily G member 8 (ABCG8) gene [8].

Our case presents a relatively young individual with very few apparent risk factors who had sudden onset of BS. However, due to the increased incidence of gallstones in the United States as stated by Unalp-Arida et al. [7], it is possible that we will begin to see BS in younger individuals. Due to this, we urge clinicians not to discount BS as a differential diagnosis in patients who may not fit the typical age or gender criteria due to the severity and potential fatality of the disease.

## Conclusions

BS is a rare type of gastric outlet obstruction secondary to a large gallstone impacted in the upper GI tract. It is often a challenging diagnosis as patients typically present with inconsistent abdominal symptoms. Our patient presented with nausea, vomiting, and abdominal pain for two days which made him visit the emergency room and eventually get evaluated by a GI specialist. BS has a high prevalence in elderly females and can be life-threatening if not recognized early. Gallstone ileus is best evaluated through CT as X-ray can be nonspecific for soft tissue imaging. The clinical presentation of abdominal symptoms guided by radiological imaging should prompt physicians to consider the possibility of BS. Even if the patient does not fit the typical age and sex, they could still be at risk for BS.
